# Structure–Activity Relationship of HER2 Receptor Targeting Peptide and Its Derivatives in Targeted Tumor Therapy

**DOI:** 10.3390/biom10020183

**Published:** 2020-01-25

**Authors:** Beáta Biri-Kovács, Afrodité Adorján, Ildikó Szabó, Bálint Szeder, Szilvia Bősze, Gábor Mező

**Affiliations:** 1 Eötvös Loránd University (ELTE), Faculty of Science, Institute of Chemistry, Budapest 1117, Hungary; biri.beata@gmail.com (B.B.-K.); adorjanafrodite@gmail.com (A.A.); 2 MTA-ELTE Research Group of Peptide Chemistry, Budapest, Hungary, Budapest 1117, Hungary; szaboi8@gmail.com (I.S.); szilvia.bosze@gmail.com (S.B.); 3 Research Centre for Natural Sciences, Institute of Enzymology, Hungarian Academy of Sciences, Budapest 1117, Hungary; szederbalint@gmail.com

**Keywords:** HER2, breast cancer, targeting peptide, targeted tumor therapy, HER2 diagnosis

## Abstract

Human epidermal growth factor (HER2) is a transmembrane tyrosine kinase receptor that is frequently overexpressed in breast cancer. Its increased level prognoses a poor patient outcome and a high mortality rate. Despite the widening spectrum of therapies that are becoming available to treat HER2+ breast cancer, its side effects and resistance still make this protein a valuable object of research in targeted tumor therapy. The role of tumor-targeting peptides has become more and more prominent in the last few decades due to their simple synthesis and pharmakokinetic properties. Here, we examine two fluorescently-labeled HER2-specific peptides and their combined analogues that are developed to target the extracellular region of HER2. The peptides are investigated on breast cancer cell lines with different HER2 expression profiles. Moreover, their extracellular localization and specificity are confirmed by flow cytometry and confocal microscopy. Therefore, a new, combined HER2 binding conjugate is obtained that interacts with HER2-overexpressing cells with high affinity and specificity. Furthermore, secondary structure prediction reveals that the α-helical content of the peptides is associated with their receptor recognition. This highly specific conjugate can be used as a starting point for diagnostical or drug-targeting purposes in upcoming studies.

## 1. Introduction

Breast cancer is still one of the leading causes of death by cancer among women worldwide. The human epidermal growth factor 2 (HER2, also called ErbB2) protein is overexpressed in 15–30% of breast cancers and prognoses an aggressive tumor type, resistance to chemotherapy, and an increased mortality rate. The *HER2* gene is also amplified in several other cancer types, such as gastric, ovarian and prostate cancers [1,2].

HER2 is a 185 kDa transmembrane glycoprotein that belongs to the epidermal growth factor receptor (EGFR) epithelial tyrosine kinase protein family, along with EGFR, HER3 and HER4. The members of the protein family possess three regions: an extracellular ligand-binding region, a single transmembrane region, and an intracellular cytoplasmic tyrosine kinase region that is responsible for signal transduction. The extracellular region consists of four domains (I–IV). The activation of the receptors occurs through the ligand-induced formation of homo- and/or heterodimers of the receptors. The only exception is HER2, which does not directly bind to any known ligands [3]. HER2 can form heterodimers with all three other members of the protein family, or, in the case of an elevated receptor concentration (such as the case in cancer), it can be found as homodimers as well. The most potent heterodimer consists of HER2 and HER3, and it is considered to be the most active signaling complex among tyrosine kinase dimers. Upon ligand binding, phosphorylation occurs and activates several downstream signaling pathways: the phosphatidyl-inositol-3 kinase (PI3K) and the Ras/Raf mitogen-activated protein kinase (MAPK) pathways. Consequently, cell proliferation, cell survival and apoptosis inhibition is enhanced [4].

Under normal circumstances, HER2 plays a vital role in mitogenic signaling, and the expression level of HER2 remains stable. However, when the overexpression of HER2 occurs, it can disrupt the dynamic balance of many cellular mechanisms and lead to uncontrollable tumor growth because: (i) Overexpression makes excessive HER2 receptors available to form extra heterocomplexes, (ii) HER2 may strengthen the affinity of ligand-binding for other receptors, (iii) HER2 might weaken the specificity of its heterodimerization partners, (iv) HER2-engaged dimerization can activate proliferation and survival, and (v) HER2-containing heterodimers may escape from the internalization or degradation of HER2 dimers. All these processes lead to increased tumorigenesis and metastasis [5,6].

Because HER2 has a crucial role in poor breast cancer prognosis, several therapies have been developed in the last decades to target this receptor. The most common strategies include the use of humanized monoclonal antibodies, small molecule tyrosine kinase inhibitors, and antibody–drug conjugates (ADCs) [1]. The first two humanized monoclonal antibodies against HER2+ breast cancer approved by the FDA (Food and Drug Administration, USA) were trastuzumab and, later, pertuzumab [7,8,9]. These antibodies bind to the extracellular domain of HER2 (trastuzumab to domain IV and pertuzumab to domain II) and, among other functions, prevent homo- and/or heterodimerization [10]. Though their use is very successful and has achieved high improvement in tumor therapy, some patients suffer from severe side effects or develop resistance [11]. Another promising therapeutic approach is the use of small molecule inhibitors (lapatinib, for example) that usually act on an intracellular level by inhibiting the downstream signaling of the cascade [12]. Several antibody–drug conjugates are also under development; ado–trastuzumab emtansine (Kadcyla)—where the cytotoxic agent is linked to trastuzumab through a thioether linker—is already approved and in use in several countries for multidrug-resistant HER2+ breast cancers [13]. This ADC prolongs the average survival rate of patients, though side effects have been reported and resistance has occurred in many cases. A relatively new approach is the design of Affibody molecules that are small (58 amino acids) antibody mimetics based on the stabilized variant of the B domain of the immunoglobulin G (IgG) binding staphylococcal protein A [14]. Affibodies have the advantage to bind specifically and with a high affinity to their target while simultaneously having a small size, making their production more affordable compared to regular antibodies. One of the most promising HER2 binding Affibody molecules is Z_HER2:342_, which interacts with domain II of the extracellular region [15]. Their possible use in human diagnostics and tumor therapy is still under clinical investigation [16]. Taken together, several therapies are available to target HER2; however, a huge number of patients still die of HER2+ breast cancer, thus calling for the identification of new agents and approaches to be used alone or in combination with existing treatments [17,18].

In clinical examination, to choose the appropriate molecular therapy and to prognose the possible outcome, HER2 status evaluation is crucial. Currently, there are several methods to detect HER2. The most frequently used test is immunohistochemistry (IHC), which is generally verified by fluorescence in situ hybridization (FISH) in the case of unambiguous cases [1]. Though IHC has its advantages (fast and relatively cheap), this semi-quantitative method also has some limitations such as the effects that are produced during sample taking (biopsies) and the lack of common guidelines for staining procedures. FISH, on the other hand, is more reliable and quantitative, although it is relatively slow and requires special expertise and the use of fluorescence microscopy [19]. Thus, the development of new, fast, low-cost and quantitative detection methods is needed.

In the last decades, tumor-targeting peptides (TTPs) have become an important field in targeted tumor therapy. Peptides have a low molecular weight, are easy to synthesize, have a high receptor recognition rate, and have better tissue penetration properties compared to ADCs. TTPs can be used as imaging agents or drug delivery systems as well [20]. Because HER2 is an ideal candidate to be the objective of targeted tumor therapy, a broad range of studies were conducted to find HER2-specific peptides. One common method for this purpose is the use of peptide bacteriophage display technology. Karasseva et al. identified a hexapeptide (KCCYSL) that most frequently occurred in an affinity-selected phage population against the extracellular domain of HER2. This peptide demonstrated a high affinity and specificity for HER2 and also bound to HER2-expressing cells [21]. Hence, we selected this peptide as one of the starting points of our study to create new, modified peptides to enhance HER2 binding affinity.

Another regularly used method to pick peptides that bind specifically to a target is molecular dynamics (MD) simulation. Geng et al. used this method to identify peptides at the extracellular domain of HER2 that are involved in dimerization. These peptides were then used to construct a “One bead one compound” (OBOC) library. After screening and sequencing, peptide affinity and specificity were analyzed. This resulted in the obtainment of 72 highly similar 17-mer peptides with a high binding affinity to the HER2 receptor [22]. The central part of these peptides was rather conservative, while the terminals showed a bit more variability. Among them, CDTFPYLGWWNPNEYRY and CKTIYYLGYYNPNEYRY showed the highest affinity to HER2 [22]. As the *N*-terminal part of the peptides (especially the latter one) showed similarities with the KCCYSL peptide, so we decided to synthesize combined peptides where the modified KCCYSL sequence precedes GYYNPN. We then compared their binding to the extracellular domain of HER2-expressing cells. Our aim was to optimize the peptide structure in a way that created specific, high affinity HER2 binding peptides that could be used in either diagnostics or in drug delivery and tumor-targeting in the future.

## 2. Materials and Methods

### 2.1. Materials

9-Fluorenylmethoxycarbonyl (Fmoc) protected amino acid derivatives and Rink amide 4-methylbenzhydrylamine (Rink amide MBHA) resin were purchased from IRIS Biotech GmbH (Marktredwitz, Germany). The reagents for synthesis were *N,N’*-diisopropylcabodiimide (DIC), N-diisopropyl-ethylamine (DIEA), 1-hydroxybenzotriazole (HOBt), 1,8-diazabicyclo-[5.4.0]undec-7-ene (DBU), piperidine, triisopropylsilane (TIS), and 5(6)-carboxyfluorescein (CF), and they were purchased from Sigma Aldrich (Budapest, Hungary). Trifluoroacetic acid (TFA) and solvents (*N,N*-dimethylformamide (DMF) and dichloromethane) (DCM)) for synthesis, as well as HPLC grade acetonitrile, were obtained from Molar Chemicals (Halásztelek, Hungary).

### 2.2. Peptide Synthesis and Purification

The synthesis of KCCYSL and GYYNPT analogue peptides was carried out on Fmoc-Rink amide MBHA resin (0.71 mmol/g) by using the Fmoc/*tert*-butyl strategy. For couplings, the carbodiimide (DIC)/HOBt or K-Oxyma Pure procedures were applied. The *N*-termini of the peptides were labeled with 5(6)-carboxyfluorescein/DIC/HOBt (3:5:5 equivalents) or were acetylated with an Ac_2_O/DIEA/DMF (1:1:3, v/v/v) mixture for 60 or 30 min, respectively. The peptides were obtained via cleavage from the resin with TFA containing D.I. water and triisopropyl silane (95:2.5:2.5) as scavengers. By this cleavage procedure, side-chain protecting groups, except for acetamidomethyl (Acm) from cysteine, residues were also removed. Crude products were precipitated by dry diethyl-ether, dissolved in a distilled water–acetonitrile mixture, and freeze-dried. The crude peptides were purified by reversed phase high performance liquid chromatography (RP-HPLC) as described below. The purified peptides were characterized by analytical RP-HPLC and electrospray ionization mass spectrometry (ESI-MS, see Supporting Information, Figures S1–S4.).

Analytical RP-HPLC was performed on a Knauer (Herbert Knauer GmbH, Berlin, Germany) HPLC system while using a Phenomenex Aeris Peptide XB-C18 column (250 × 4.6 mm I.D.) with 3.6 µm silica (Torrance, CA USA) as a stationary phase. A linear gradient elution (0 min 0% B; 5 min 0% B; 50 min 90% B) with eluent A (0.1% TFA in water) and eluent B (0.1% TFA in acetonitrile–water (80:20, *v*/*v*)) was used at a flow rate of 1 mL/min at ambient temperature. Peaks were detected at λ = 220 nm. The samples were dissolved in eluent B.

The crude products were purified on a semipreparative Inertsil^®^ ODS-3 C18 column (250 × 10mm I.D.) with 10 µm silica (GL Sciences, Tokyo, Japan). The flow rate was 4 mL/min. Linear gradient elution was applied.

The identification of the products was achieved by mass spectrometry. ESI-MS was performed on Bruker Esquire 3000 Plus Ion Trap Mass Spectrometer (Bremen, Germany) that operated in continuous sample injection at a 10 µL/min flow rate. Samples were dissolved in 50% acetonitrile–50% water containing 0.1% acetic acid. Mass spectra were recorded in positive mode in the *m/z* 50–2000 range.

### 2.3. Cell Culturing

Human epithelial breast cancer cell lines (derived from metastatic sites)—MDA-MB-453 (American Type Culture Collection, ATCC^®^ HTB-131™; pericardial effusion, metastatic carcinoma) [23,24] and MDA-MB-435 Brain (MDA-MB-435 Br—were a generous gift from Professor Angels Fabra. Pleural effusion, adenocarcinoma [25,26,27]) and MCF-7 (ATCC^®^ HTB22™; pleural effusion, adenocarcinoma) [28,29] were maintained as adherent cultures in Dulbecco’s Modified Eagle Medium (DMEM, Lonza™) supplemented with 10% heat-inactivated fetal calf serum (FCS, Lonza™), l-glutamine (2 mM) and a penicillin–streptomycin antibiotics mixture (Lonza™ BioWhittaker™, 5000 IU/5000 IU). HCC1143 breast epithelial carcinoma cells that were derived from an invasive ductal carcinoma (ATCC^®^ CRL-2321™) were kindly provided by József Tóvári (National Institute of Oncology, Budapest, Hungary) and maintained in Roosevelt Park Memorial Institute RPMI-1640 that was supplemented with 10% FCS, l-glutamine and penicillin–streptomycin antibiotics mixture. All cell lines were grown at 37 °C in a humidified atmosphere containing 5% CO_2_.

### 2.4. Western Blot

Cells were lysed in a lysis buffer containing 50 mM Tris pH 7.4, 150 mM NaCl, 2 mM ethylenediaminetetraacetic acid (EDTA), 1% Triton-X 100 and a Halt^TM^ Protease Inhibitor Cocktail (100x, Thermo Fisher Scientific, Waltham, MA, USA). Protein concentration was measured by a Qubit^TM^ Protein Assay Kit (Thermo Fisher Scientific), according to the manufacturer’s instructions. Equal amount of proteins was run on 10% Tris-tricine gel and blotted to polyvinylidene fluoride (PVDF) membrane with a Bio-Rad Wet Blotting System (Bio-Rad Hungary, Budapest, Hungary). The HER2 receptor was detected by an ErbB2 (HER2) antibody cocktail (Thermo Fisher Scientific, MA5-14057, produced in mouse, 1:500) and an anti-mouse-horseradish peroxidase (HRP) secondary antibody (Thermo Fisher Scientific, 32430, produced in goat, 1:500). As a loading control, β-actin was detected by an anti-actin antibody (Santa Cruz Biotechnology, Dallas, TA, USA, sc-1616, produced in goat, 1:2500) and an anti-goat-HRP secondary antibody (Santa Cruz Biotechnology, sc-2354, produced in mouse, 1:2000). After the addition of enhanced chemiluminescence (ECL) substrate (SuperSignal^TM^ West Pico PLUS Chemiluminescent Substrate, Thermo Fisher Scientific), the chemiluminescent signal was detected by ChemiDoc XRS+ Detection System (Bio-Rad Hungary).

### 2.5. In Vitro Flow Cytometry Evaluation and Fluorescent Microscopy

The internalization and HER2 specificity of the CF-labeled peptides were measured by using a Beckton-Dickinson (BD) LSR II flow cytometer (BD Biosciences, San Jose, CA, USA) with a 488 nm (Coherent Sapphire, 22 mW) laser. Cells were seeded to 24-well plates (Sarstedt, Nümbrecht, Germany)) at a density of 10^5^ cells/well. The next day, cells were treated with peptides at different concentrations and were incubated for 1 or 3 h (37 °C, 5% CO_2_ atmosphere). In the case of the blocking experiment, cells were preincubated with unlabeled peptides (65 µM) for 30 min before the addition of the CF-labeled peptide (12.5 or 6.25 µM, 3 h). After centrifugation (1000 rpm, 5 min) and washing with serum-free DMEM, the supernatant was removed and 100 μL of 0.25% trypsin (Sigma-Aldrich) was added to the cells. To block trypsin activity, after 5 or 15 min of incubation, 0.8 mL of a 10% FCS/Hepes-buffered HPMI medium was added, and then cells were washed and re-suspended in a 0.3 mL HPMI medium (that contained 9 mM glucose, 10 mM NaHCO_3_, 119 mM NaCl, 9 mM 4-(2-hydroxyethyl)-1-piperazineethanesulfonic acid (HEPES), 5 mM KCl, 0.85 mM MgCl_2_, 0.053 mM CaCl_2_, 5 mM Na_2_HPO_4_ × 2H_2_O, and pH 7.4 [30] that were prepared in our laboratory by using components obtained from Sigma-Aldrich). The fluorescence intensity of the cells was measured on a BD LSR II, channel FITC LP505 (emission at λ = 505 nm; LP 505, BP 530/30), and the data were analyzed with the FACSDiva 5.0 software. All measurements were performed in duplicates, and the mean fluorescent intensity and the standard error of the mean (SEM) are graphically presented.

Parallel to the flow cytometry measurements, to visualize cell morphology after CF-peptide treatment, fluorescent microscopic images were captured. Washed and resuspended cells were plated in a 96-well flat bottom tissue culture plate, and images of the adherent cells were captured by using an Olympus CKX41 microscope (Hamburg, Germany, Olympus U-RFLT50 mercury-vapor lamp, WideBlue DM500 BP460-490 BA520 IF filter).

### 2.6. Immunostaining and Confocal Microscopy

Cells were seeded to coverslip-containing (thickness 1, Assistant, Karl Hecht GmbH & Co KG, Sondheim/Rhön, Germany) 24-well plates (Sarstedt) one day prior to treatment at a density of 5 × 104 cells/well. Cells were treated with 25 µM CF-conjugated peptides that were diluted in a serum-free medium for 90 min. For blocking studies, cells were preincubated with unlabeled peptides without CF conjugation (65 µM, 30 min). In this case, the CF-conjugated peptides were used at a 12.5 µM concentration. After washing with phosphate buffered saline (PBS, Lonza, Basel, Switzerland), cells were fixed with 4% paraformaldehyde for 20 min at 37 °C. Permeabilization and blocking were performed in 3% bovine serum albumin (BSA, Sigma-Aldrich) that was dissolved in PBS and contained 0.3% Triton-X 100 for 1 h at room temperature. The HER2-receptor was detected by the ErbB2 (HER2) antibody cocktail (see above, 1:200, at 4 °C, overnight), and a tetramethylrhodamine (TRITC)-conjugated anti-mouse antibody was used as a secondary antibody (Thermo Fisher Scientific, A16071, produced in goat, 1:250, 1 h, at room temperature). The antibodies were diluted in PBS that contained 1% BSA and 0.1% Triton-X 100. Nuclei were stained with DAPI (4′,6-diamidino-2-phenylindole, Sigma-Aldrich, 0.2 µg/mL diluted in PBS, for 15 min at room temperature). Coverslips were mounted to microscopy slides by using a Mowiol^®^ 4–88 mounting medium (Sigma-Aldrich). Confocal microscopy images were acquired on a Zeiss LSM 710 system (Carl Zeiss Microscopy GmbH, Jena, Germany) with a 40X oil objective. ZEN Lite (Carl Zeiss Microscopy GmbH) software was used for image processing.

### 2.7. Secondary Structure Estimation

The secondary structure of the conjugates was estimated by the PEP-FOLD3 de novo peptide structure prediction server [31,32].

## 3. Results

### 3.1. Peptide Synthesis

After selecting the sequence of KCCYSL for HER2 targeting [21], we designed some further analogues to investigate the role of the two central cysteine residues. First, the role of the distance of the two amino acids was observed by introducing glycine residues between them. Subsequently, the importance of the disulfide bond forming capacity was investigated by using Acm protection groups on both cysteines. Finally, cysteines were systematically replaced by serine or alanine.

The other chosen peptide (GYYNPN), derived from the MD simulation and refined by creating an OBOC library [22], was also measured alone or in combination with the first sequence. Here, in order to prevent succinimide formation, we replaced the last amino acid of the sequence with threonine, which, in this position, was the second most frequent amino acid after asparagineamong the 72 verified positive sequences [22]. The corresponding sequences and codes can be seen in Table 1. The peptides were prepared by solid phase peptide synthesis on Rink amide MBHA resin by using the Fmoc/tBu protocol. In the last step, 5(6)-carboxyfluorescein (CF) as fluorescent dye was attached to the *N*-terminus of the peptidyl resins. The compounds were analyzed by analytical RP-HPLC, and their molecular weight was identified by ESI-MS (Table 1). The results obtained by RP-HPLC and ESI-MS measurements can be seen in the Supplementary information (Figures S1–S4).

### 3.2. In Vitro Biological Activity

Based on histopathological and molecular biological considerations, breast carcinomas are divided into five major subtypes: Luminal A, luminal B, Basal, HER2-enriched and normal-like subtypes. While the first two are eligible for hormone therapies, HER2+-positive cancers require different treatments. During the development and testing of new compounds, the data on the expression level of the targeted molecule are fundamental [33].

Information about the HER2 status of commonly used human breast cancer cell lines has shown great variations in the literature. Though choosing the right cell line for testing new compounds is crucial, we found confusing facts when we tried to look up a particular cell line: Often, cells are considered low-expressing in one work and overexpressing in a different study [34]. This can have multiple reasons from different detection techniques and classification systems to the heterogeneity of cell lines (and culture conditions) in laboratories. Thus, evaluating target protein levels before choosing the subject of one’s research is of great importance [35,36].

**Figure 1:**
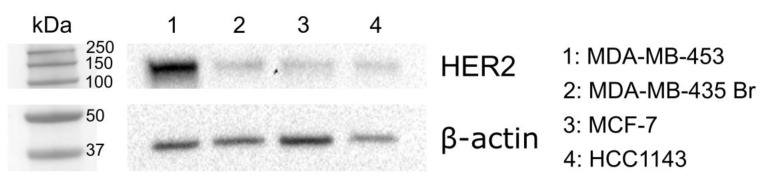
Western blot analysis of HER2 expression level of different cell lines. Equal protein amounts were run on Tris-tricine gel and blotted to PVDF membrane. As loading control, β-actin was used.

To assess the HER2 expression level of different breast cancer cell lines that are derived from metastatic sites, western blot was performed by using an anti-HER2 antibody. By comparing expression levels, we could detect the highest HER2 expression in MDA-MB-453 cells, while MDA-MB-435 Br and MCF-7 cells showed a medium expression. As a negative control, HCC1143, which is a breast cancer cell line that is derived from an invasive ductal carcinoma with no lymph node metastases, was used. This cell line expressed the lowest HER2 levels according to the results of the western blot (Figure 1).

Consequently, the in vitro biological activity of synthetic compounds was first measured on MDA-MB-453 cells (derived from pericardial effusion) by flow cytometry. The viability of the cells was generally above 70–80%, and we did not observe major cell death in the case of peptide treatment (for data concerning mean viability in flow cytometry experiments; see Tables S1 and S2 and S3). This observation was in accordance with microscopic images where dramatic changes in cell morphology were not detected upon treatment with CF-peptides.

**Figure 2:**
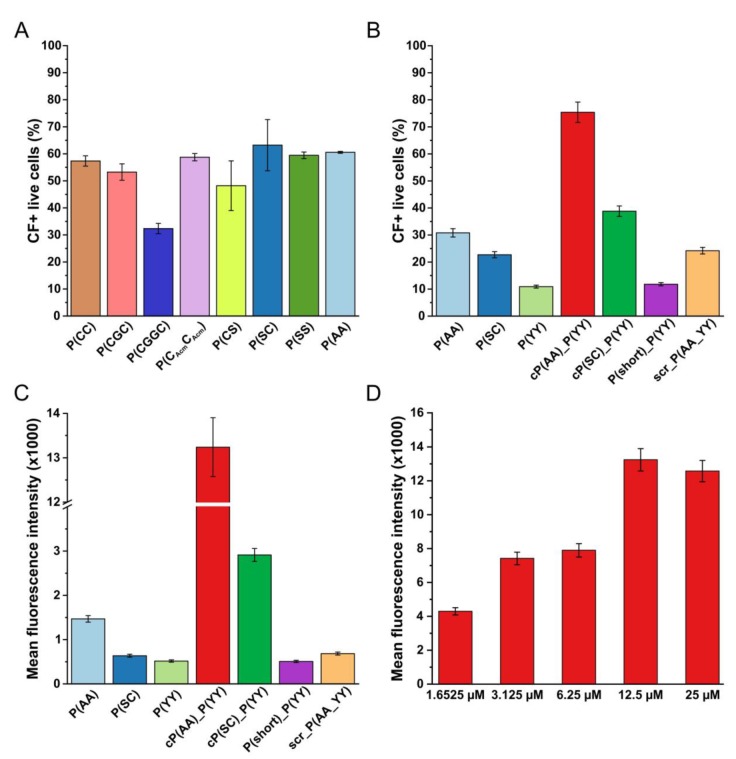
Cellular uptake profile of 5(6)-carboxyfluorescein (CF)-labeled HER2 binding peptides using MDA-MB-453 breast cancer cells. (**A**) Comparison of the first set of peptides using a 1 h incubation time and a concentration of 100 µM. (**B,C**) Cellular uptake (percentage of CF+ live cells and mean fluorescence intensity) of the most promising conjugates from the first set of peptides, the peptide chosen from the molecular dynamics (MD) simulation and “one bead one compound” (OBOC) library and combined peptides using 3 h incubation time and a concentration of 12.5 µM. (**D**) Concentration dependence of cellular uptake of conjugate cP(AA)_P(YY), mean fluorescence intensity after 3 h of incubation. Flow cytometry was measured by a BD LSR II flow cytometer. The mean and standard deviation of two parallels are depicted. The experiments were repeated twice.

First, the members of the first set of compounds (P(CC) and its variants) were compared. The results showed that introducing additional amino acids (glycines) between the two cysteine residues decreased the percentage of CF+ cells. Moreover, as the introduction of the acetamidomethyl group did not change the cellular uptake, we can conclude that the disulfide bond formation ability of the corresponding cysteine residues did not contribute to their possible HER2 binding capacity. On the contrary, the replacement of cysteines with serines one by one or changing both of them to two serines or alanines resulted in an increased cellular uptake with the exception of the P(CS) compound (Figure 2A). As the P(SC) and the P(AA) compounds showed the highest uptake in this set, we included these two in further studies and during the design of the combined peptides.

Afterwards, the two most promising compounds were examined in contrast with the compound chosen from the MD simulation and OBOC library (P(YY)). Here, we also added combined versions to the comparison, namely cP(AA)_P(YY) and cP(SC)_P(YY), along with a shorter combined version not including the first three amino acids (P(short)_P(YY)). As a negative control, the scrambled version of one of the combined peptides (scr_P(AA_YY)) was used. The flow cytometry results showed that less P(YY) was uptaken as compared to P(AA) and P(SC). However, both combined compounds showed an elevated cellular uptake. The P(short)_P(YY) compound was uptaken only at a level similar to the P(YY) peptide. Interestingly, the scrambled negative control also showed some uptake, though the values were lower than the two longest combined ones (Figure 2B). Along with the percentage of CF+ live cells, the mean fluorescence intensity was also investigated, resulting in higher differences, especially in the case of the most promising cP(AA)_P(YY) compound, where a roughly tenfold increase was observed compared to the P(AA) compound (Figure 2C). As a consequence, in further experiments, the mean fluorescence intensity values were compared. With an increased incubation time (3 vs. 1 h) the differences were more remarkable; thus, we used a 3 h incubation time in consequent flow cytometry measurements. The cellular uptake of the conjugates was measured at different concentrations. Here, the concentration-dependent cellular uptake of cP(AA)_P(YY) was shown, because we could observe that by increasing the amount of the conjugate, the cellular uptake was enhanced until 12.5 µM, after which a saturation could be observed (Figure 2D). A comparison of the concentration-dependent cellular uptake of the tested peptides can be seen in Figure S5.

### 3.3. Cellular Localization

**Figure 3:**
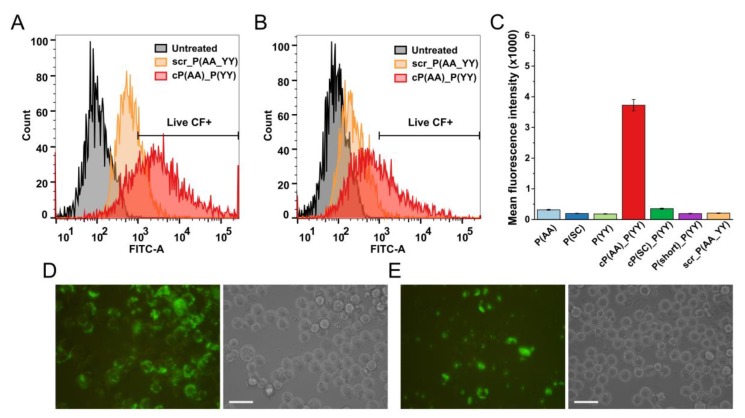
Effect of increased trypsinization time on cellular uptake of CF-labeled peptides by MDA-MB-453 cells. (**A**,**B**) Histogram of cellular uptake followed by 5 and 15 min of trypsinization, respectively. (**C**) Mean fluorescence intensity after 15 min of trypsinization. Cells were incubated with the conjugates (12.5 µM) for 3 h. Flow cytometry was measured by a BD LSR II flow cytometer. The mean and standard deviation of two parallels are depicted. The experiments were repeated twice. (**D,E**) Cellular uptake captured by fluorescence microscopy after 5 and 15 min of trypsinization, respectively. Scale bars represent 50 µm.

As both initial HER2 peptides were selected on the extracellular domain of HER2, we went on to detect the conjugates at the plasma membrane. The cellular localization of the CF-labeled peptides was first investigated via extended trypsinization. In the case of MDA-MB-453 cells, five min of trypsinization was generally used to detach the cells prior to flow cytometry measurement. With an elevated incubation time, the possible extracellular localization of the peptides was monitored. Using 15 min of trypsinization indeed decreased the measured fluorescence intensity values (Figure 3A–C) without decreasing the viability of the cells (Figure S7A,C) The decreased signal was also captured by fluorescence microscopy: By extending the incubation time with trypsin, a lower fluorescence signal was detected (Figure 3D,E).

**Figure 4:**
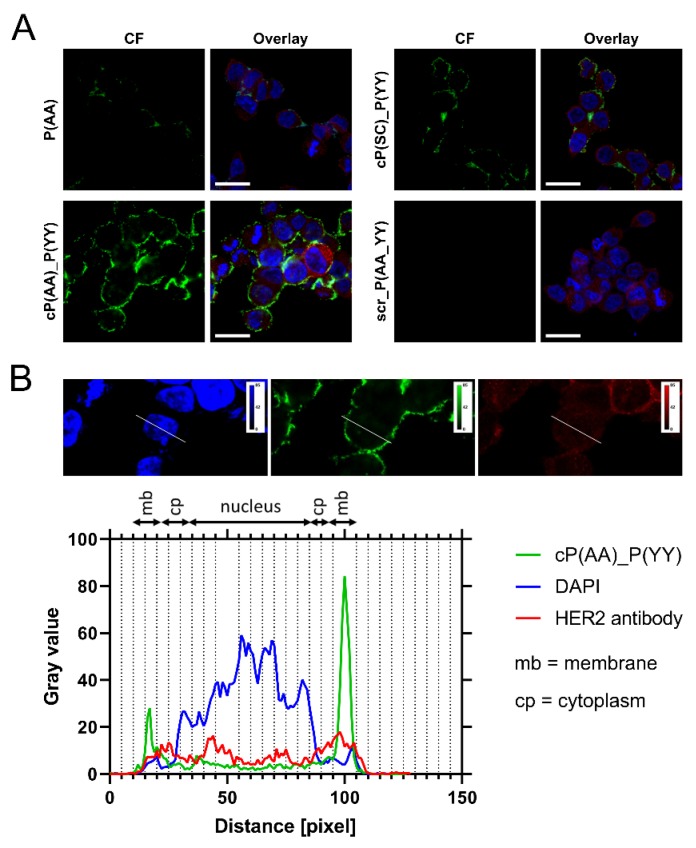
Cellular localization of peptides in MDA-MB-453 cells. (**A**) Cellular uptake of peptides P(AA), cP(AA)_P(YY), cP(SC)_P(YY) and scr_P(AA_YY) studied by confocal microscopy. Cells were incubated with CF-labeled peptides (25 µM) for 90 min (green). HER2 was detected with an anti-HER2 antibody and a TRITC-conjugated secondary antibody (red). Nuclei were stained with 4’6-diamidino-2-phenylindole (DAPI, blue). Zeiss LSM 710 system was used for image acquisition. Scale bars represent 20 µm. (**B**) Line scan of the MDA-MB-453 cells conducted along the white line overlaying the images of the fluorescent channels (top). Green shows the localization of the cP(AA)_P(YY) peptide (below), and blue and red correspond to DAPI and HER2 signals, respectively.

The cellular localization of the CF-conjugated peptides was also monitored by confocal microscopy. Beside carboxyfluorescein, HER2 was also visualized by an HER2-specific antibody. In accordance with the fluorescence microscopy data, peptides localized mainly at the cell membrane and could only be seen in the cytoplasm in small quantities. We could detect the highest signal in case of the conjugate cP(AA)_P(YY), followed by cP(SC)_P(YY). The P(AA) conjugate showed a medium cellular uptake, while the signal from the negative control scr_P(AA_YY) (Figure 4A), along with peptides P(SC), P(YY) and P(short)_P(YY) (Figure S6), could not be detected. A line scan of images of the cP(AA)_P(YY) uptake captured by confocal microscope also showed plasma membrane localization, while the peptide quantities in the cytoplasm and nucleus were low (Figure 4B).

### 3.4. Cellular Uptake in Cells with Lower HER2 Expression

Subsequently, cellular uptake was also measured on cell lines with lower HER2 expression. MDA-MB-435 Br is a brain metastasis of the breast cancer cell line MDA-MB-435. This cell line, along with the widely used MCF-7 breast cancer cells, showed a medium HER2 expression (Figure 1). The HER2 expression of HCC1143 was under our detection limit, so we used these cells as a negative control.

**Figure 5:**
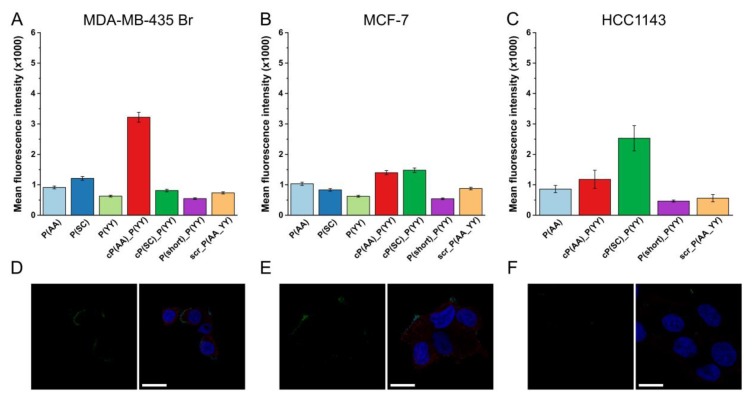
Cellular uptake of HER2-specific compounds by cell lines with a lower HER2 expression. (**A,B,C**) Flow cytometry analysis of the HER2 binding compounds (12.5 µM) on cell lines with a medium or lower HER2 expression after 3 h incubation. Flow cytometry was measured by a BD LSR II flow cytometer. The mean and standard deviation of two parallels are depicted. The experiments were repeated twice. (**D,E,F**) Confocal microscopic imaging of cP(AA)_P(YY) (25 µM) after 90 min incubation is shown in green. HER2 was detected with the anti-HER2 antibody and the TRITC-conjugated secondary antibody (as shown in red). Nuclei were stained with DAPI (blue). The Zeiss LSM 710 system was used for image acquisition. Scale bars represent 20 µm.

The flow cytometry results demonstrated that the cell lines showing a lower HER2 expression were able to uptake the conjugates at a lower rate, especially in case of the most promising cP(AA)_P(YY) combined conjugate (Figure 5 A–C). The cellular uptake by the MDA-MB-435 Br cells using a prolonged trypsinization resulted in lower fluorescence intensity values (Figure S7B,D). The cellular localization of the conjugates was also analyzed. Analyzing the cell lines that showed a medium HER2 expression resulted in the detection of lower signals at the cell membrane, while in case of the cell line showing the lowest HER2 expression (HCC1143), almost no signal could be seen (Figure 5 D–F, S8 and S9).

### 3.5. Receptor Specificity of HER2-Binding Peptides

**Figure 6:**
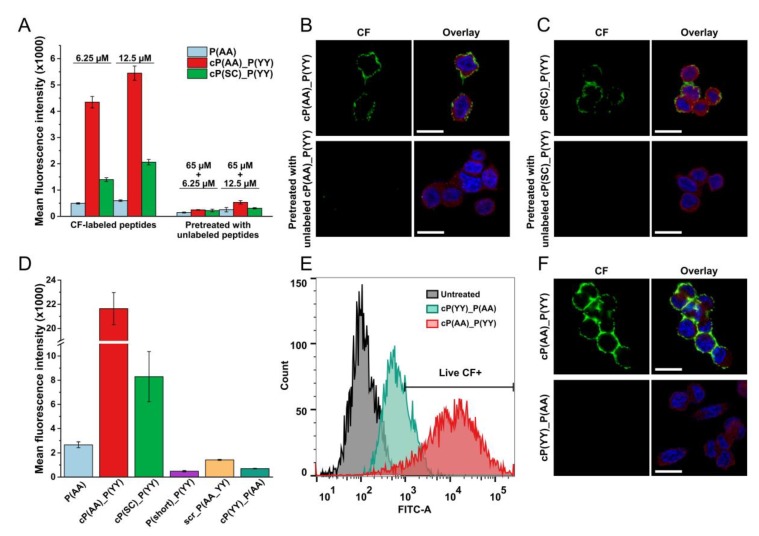
Specificity study of HER2-binding compounds. (**A**) MDA-MB-453 cells were preincubated with unlabeled peptides, and fluorescence was measured by flow cytometry. (**B**,**C**) Confocal microscopic imaging of conjugates cP(AA)_P(YY) and cP(SC)_P(YY), respectively, which were pretreated with unlabeled peptides. (**D**,**E**) Analysis of the reversed combined peptide (cP(YY)_P(AA)) by flow cytometry. (**F**) Imaging of the reversed combined peptide by confocal microscopy. The CF signal and the nucleus staining are represented by green and blue, respectively. HER2 was detected with an anti-HER2 antibody and a TRITC-conjugated secondary antibody, as shown in red. A Zeiss LSM 710 system was used for image acquisition. Scale bars represent 20 µm.

To study the specificity of the peptides, a blocking experiment that used unlabeled peptides was performed, and the two peptides that showed the highest cellular uptake were cP(AA)_P(YY) and cP(SC)_P(YY). First, the cells were preincubated with a five-times molar excess of unlabeled peptides, which was followed by incubation with the corresponding CF-labeled peptide. The flow cytometry results showed that the addition of unlabeled peptides prior to CF-labeled compounds decreased the detected CF-signal in both cases (Figure 6A). These results were also confirmed by confocal microscopy, as the unlabeled peptides inhibited the binding of the CF-labeled peptides (Figure 6B,C).

To analyze the specificity of the binding of cP(AA)_P(YY), a reversed compound was also designed wherein the sequence order of KAAYSL and GYYNPT was swapped (resulting in the compound cP(YY)_P(AA)). This reversed compound showed a low-affinity binding in flow cytometry measurements and a decreased signal in microscopy studies (Figure 6D,F).

### 3.6. Secondary Structure Prediction

**Figure 7:**
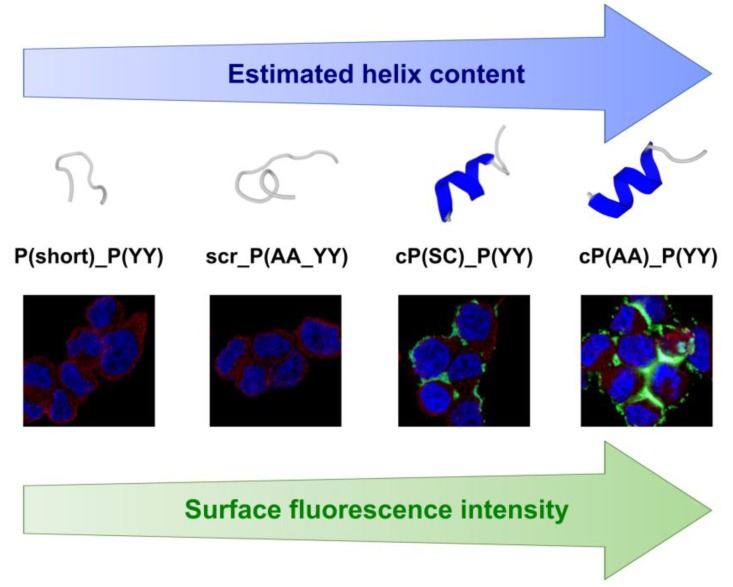
Estimated α-helical content of the HER2-binding peptides is proportional to the fluorescence intensity that was detected on the surface of the MDA-MB-453 cells that were incubated with the CF-labeled peptides. Secondary structure estimation of the peptides was performed by the PEP-FOLD3 de novo secondary structure prediction server. The color grey shows disordered regions, and thepredicted α-helical content is marked with blue. Cell imaging was carried out by confocal microscopy where the CF signal and nucleus staining are represented by green and blue, respectively. HER2 was detected with an anti-HER2 antibody and a TRITC-conjugated secondary antibody, as shown in red.

The PEP-FOLD3 algorithm was used [37,38,39] to make an estimation on the secondary structure of the peptides. The structural results of the PEP-FOLD algorithm and a predicted 3D conformation are shown in Figure 7. The PEP-FOLD results can be interpreted as the probability of an amino acid in the sequence to be in a helical, random coil, or an extended strand structure.

According to the structural information that was provided by the application, the P(short)_P(YY) peptide is mostly a random coil and does not form a helical structure at all, while the probability of helix formation is quite low for the peptide with the scrambled sequence scr_P(AA_YY). On the other hand, helix formation is highly feasible for peptides cP(AA)_P(YY) and cP(SC)_P(YY), and their binding ability is high.

## 4. Discussion

The importance of HER2, as one of the most promising cell surface molecules for diagnostic imaging and targeted therapy in breast cancer, is indisputable. Despite the numerous in vitro and in vivo studies that have described the involvement of HER2 in proliferation, survival, and metastasis induction, some cellular behaviors remain unexplained, and new approaches to treat HER2+ breast cancer are needed [40,41]. Besides the use of monoclonal antibodies and small molecule inhibitors, the role of tumor-targeting peptides is emerging for both diagnostical and drug delivery aspects.

Here, we examined two different HER2-targeting peptides that were derived from preceding studies, modified their sequence, and selected the ones that were able to bind at the highest rate and most specifically to HER2-overexpressing breast cancer cell lines. The hexapeptide that was derived from the bacteriophage display library (KCCYSL) [21] was replaced with two analogs for further studies (KAAYSL and KSCYSL), as these two conjugates displayed higher cellular uptake values in flow cytometry experiments. As P(AA) and P(SC) had smaller cysteine values (or none), these might be more favorable in terms of avoiding disulfide bond formation and possibly resulting in dimer peptides. Larimer et al. attempted to improve the binding of the KCCYSL peptide to HER2 by prolonging it towards both the *N*- and *C*-terminal regions (with five and four amino acids, respectively), though no enhanced binding affinity (or only modest improvements) could be detected [42]. Hence, we decided to elongate P(AA) and P(SC) in the *C*-terminal direction with more amino acids. A certain similarity could be observed by analyzing HER2 binding sequences (derived from an MD simulation and OBOC library screening [22]) This resulted in the formation of combined peptides composed of 12 amino acids.

In contrast to the CF-labeled hexapeptides, the fluorescent signal after the treatment with designed combined peptides cP(AA)_P(YY) and cP(SC)_P(YY) was increased. The mean fluorescence intensity values showed a ten-fold enhancement compared to all shorter hexapeptides, thus implicating a synergistic effect between the two initial sets in terms of binding affinity. The localization of these CF-labeled combined peptides was mainly not intracellular, but they were detected on the cell membrane by fluorescent and confocal microscopy. The extracellular localization was also emphasized by trypsinization that resulted in a significantly lower fluorescence intensity. Our data suggest that the combined peptide is indeed suitable for receptor binding because it has a high affinity, and the internalization rate of the peptides is low.

The specificity studies included cellular uptake studies on cell lines with lower HER2 expression levels. The results indicate that the HER2 expression profile is related to the binding of the conjugates. Moreover, the pretreatment of cells with unlabeled peptides of the same sequence confirm that these conjugates bind to a particular extracellular protein on the surface of breast cancer cells. The results of the reversed combined peptide where the KAAYSL sequence is at the *C*-terminal region is preceded by GYYNPT confirmed that the position of the amino acids is of great importance for the interaction.

The prediction of the secondary structure revealed a certain correspondence between the predicted alpha-helical content of the peptides and their binding affinity. Here, it has to be stated that the reliability of secondary structure prediction in case of such short peptides is still lower compared to longer ones [32]. Furthermore, short peptides mostly reside in a disordered state or switch between alternative conformations in solution, and they gain their final structure only upon binding to their interaction partners [43]. However, because of the possible importance of alpha helices in the peptides, we compared the sequence of cP(AA)_(YY) with a known alpha helical HER2 interaction partner, Z_HER2:342_, an HER2 binding Affibody molecule consisting of three helices [15]. The comparison revealed a certain resemblance with the Affibody at the region starting from lysine at position 27 (KRAFIRSLYDDPS vs. KAAYSLGYYNPT). In addition, this region in the Affibody molecule contains key residues for HER2 binding [15,44]. Hence, the exact interaction domain between HER2 and cP(AA)_(YY) and a possible overlapping with the Affibody binding site need to be determined by structural studies of the complex. Revealing the localization of the involved binding and amino acids could lead to the optimization of the length of the peptide and a base to include different residues that improve the binding affinity.

The further aim of our study is to possibly apply the selected combined homing peptides for tumor diagnostics (e.g., positron-emission tomography) or the selective delivery of radiotracers with therapeutic activity. In addition, the application of extracellular enzyme (elastase, matrix metalloproteinases) cleavable spacers between the homing peptide and an antitumor agent might be a good choice for the development of conjugates as drug delivery systems.

## 5. Conclusions

Since HER2 is an important target in breast cancer treatment, there is an emerging number of therapeutics and possible diagnostic tools that target this transmembrane receptor. One of the possible approaches is the development of tumor-targeting peptides. In our study, we proceeded from two peptides that are known to bind to the extracellular region of HER2. By generating different analogues and combining the two sets, a new 12 amino acid long peptide that binds to HER2-overexpressing breast cancer cells with high affinity and specificity was revealed. Microscopical experiments validated the extracellular localization of the compounds, and their specificity was verified by the preincubation of cells with unlabeled peptides. Secondary structure prediction revealed the possible importance of the α-helical structure of the conjugates. As a consequence, this homing peptide with a high affinity and specificity for the HER2 extracellular domain can be applied in further studies for tumor diagnostics and drug targeting.

## Figures and Tables

**Table 1:** List of HER2 binding compounds with their corresponding codes, RP-HPLC retention times, and their calculated and measured molecular weight.

Compound	Code	RP-HPLC R_t_ (min) ^1^	ESI-MS MW (calculated)	ESI-MS MW (measured) ^2^
CF-KCCYSL-NH_2_	P(CC)	29.9^a^	1072.9	1072.3
CF-KCGCYSL-NH_2_	P(CGC)	29.4^a^	1129.9	1129.8
CF-KCGGCYSL-NH_2_	P(CGGC)	31.7^a^	1186.0	1187.0
CF-KC_(Acm)_C_(Acm)_YSL-NH_2_	P(C_(Acm)_C_(Acm)_)	28.8^a^	1214.5	1214.0
CF-KCSYSL-NH_2_	P(CS)	29.7^a^	1056.7	1056.4
CF-KSCYSL-NH_2_	P(SC)	29.5^a^	1056.7	1056.4
CF-KSSYSL-NH_2_	P(SS)	27.7^a^	1040.8	1040.5
CF-KAAYSL-NH_2_	P(AA)	28.8^a^	1007.8	1008.4
CF-GYYNPT-NH_2_	P(YY)	30.0^a^	1070.8	1070.4
CF-KAAYSLGYYNPT-NH_2_	cP(AA)_P(YY)	21.4^b^	1704.5	1704.6
CF-KSCYSLGYYNPT-NH_2_	cP(SC)_P(YY)	21.4^b^	1751.8	1752.2
CF-YSLGYYNPT-NH_2_	P(short)_P(YY)	22.7^b^	1433.8	1433.8
CF-TAKLYPGYANYS-NH_2_	scr_P(AA_YY)	21.1^b^	1704.5	1704.0
CF-GYYNPTKAAYSL-NH_2_	cP(YY)_P(AA)	21.6^b^	1704.5	1704.6
H-KAAYSLGYYNPT-NH_2_	Unlabeled cP(AA)_P(YY)	19.4^b^	1345.7	1346.0
H-KSCYSLGYYNPT-NH_2_	Unlabeled cP(SC)_P(YY)	19.4^b^	1393.6	1394.0

^1^ RP-HPLC: ^a^: column: Phenomenex Aeris Peptide XB-C18 column (250 × 4.6 mm) with 3.6 μm silica; eluents: 0.1% TFA in water (A) and 0.1% TFA in acetonitrile–water (80:20, *v*/*v*) (B); gradient: 0 min 0% B, 5 min 0% B, 50 min 90% B; flow rate: 1 mL/min; detection: λ = 220 nm, ^b^: column: Macherey–Nagel Nucleosil C18 column (250 × 4.6 mm) with 5 µm silica (100 Å pore size); eluents: 0.1% TFA in water (A) and 0.1% TFA/acetonitrile −water (80:20 *v*/*v*) (B); gradient 0 min 2% B, 5 min 2% B, 30 min 90% B; flow rate 1 mL/min; detection: 220 nm. ^2^ ESI-MS: Esquire 3000+ ion trap mass spectrometer (Bruker Daltonics). Code for individual peptide: peptide (P) and in bracket the one letter symbol of amino acids in the variable positions; c: combined sequence and scr: scrambled derivative.

## Supplementary Materials

The following are available online at https://www.mdpi.com/2218-273X/10/2/183/s1.
