# Supplementary material for: Structure–Activity Relationship of HER2 Receptor Targeting Peptide and Its Derivatives in Targeted Tumor Therapy
Source: Biomolecules. 2020 Jan 25;10(2):183. doi: 10.3390/biom10020183 (PMC7072344; doi:10.3390/biom10020183)
Supplement: Supplementary file 1 [file biomolecules-10-00183-s001.pdf]

Structure-Activity Relationship of HER2 Receptor Targeting Peptide and Its Derivatives in Targeted Tumor Therapy

Beáta Biri-Kovács[1,2,†], Afrodité Adorján[1,2,†], Ildikó Szabó[2], Bálint Szeder[3], Szilvia Bősze[2] and Gábor Mező[1,2*]

[1] Eötvös Loránd University (ELTE), Faculty of Science, Institute of Chemistry, Budapest, 1117, Hungary
[2] MTA-ELTE Research Group of Peptide Chemistry, Budapest, Hungary, Budapest, 1117, Hungary
[3] Research Centre for Natural Sciences, Institute of Enzymology, Hungarian Academy of Sciences, Budapest, 1117, Hungary
* Correspondence: gmezo@elte.hu; +3613722500
† These authors contributed equally to this work.

Supplementary information.

Contents

Table S1.	3
Table S2.	4
Figure S1.	5
Figure S2.	6
Figure S3.	7
Figure S4.	8
Figure S5.	9
Figure S6.	10
Figure S7.	11
Figure S8.	12
Figure S9.	13

Table S1. Mean viability (%) data recorded in flow cytometry experiments.

Sample name (Fig. 2A)	Mean viability (%)
Control	88.3
P(CC)	88.2
P(CGC)	88.4
P(CGGC)	86.4
P($C_{(Acm)}C_{(Acm)}$)	87.1
P(CS)	88.9
P(SC)	86.4
P(SS)	88.4
P(AA)	87.8

Sample name (Fig. 2B)	Mean viability (%)
Control	59.4
P(AA)	63.7
P(SC)	69.2
P(YY)	63.0
cP(AA)_P(YY)	64.2
cP(SC)_P(YY)	60.4
P(short)_P(YY)	63.2
scr_P(AA_YY)	61.1

Sample name (Fig. 3C)	Mean viability (%)
Control	68.1
P(AA)	70.4
P(SC)	79.0
P(YY)	77.3
cP(AA)_P(YY)	75.1
cP(SC)_P(YY)	77.5
P(short)_P(YY)	66.3
scr_P(AA_YY)	71.9

Sample name (Fig. 5A)	Mean viability (%)
Control	85.5
P(AA)	88.8
P(SC)	86.1
P(YY)	86.5
cP(AA)_P(YY)	87.7
cP(SC)_P(YY)	87.0
P(short)_P(YY)	84.7
scr_P(AA_YY)	88.8

Sample name (Fig. 5B)	Mean viability (%)
Control	76.2
P(AA)	81.1
P(SC)	77.3
P(YY)	80.3
cP(AA)_P(YY)	80.8
cP(SC)_P(YY)	86.6
P(short)_P(YY)	87.3
scr_P(AA_YY)	85.8

Sample name (Fig. 5C)	Mean viability (%)
Control	90.0
P(AA)	92.9
P(YY)	92.9
cP(AA)_P(YY)	92.9
cP(SC)_P(YY)	93.0
P(short)_P(YY)	93.2

Sample name (Fig. 6A)	Mean viability (%)
Control	87.0
P(AA) (a)	82.3
P(AA) (b)	83.0
P(AA) (c)	85.5
P(AA) (d)	76.0
cP(AA)_P(YY) (a)	82.3
cP(AA)_P(YY) (b)	84.2
cP(AA)_P(YY) (c)	84.9
cP(AA)_P(YY) (d)	81.8
cP(SC)_P(YY) (a)	94.2
cP(SC)_P(YY) (b)	93.5
cP(SC)_P(YY) (c)	91.8
cP(SC)_P(YY) (d)	92.8

Sample name (Fig. 6D)	Mean viability (%)
Control	87.0
P(AA)	87.1
P(YY)	81.1
cP(AA)_P(YY)	82.9
cP(SC)_P(YY)	80.5
P(short)_P(YY)	77.1
cP(YY)_P(AA)	75.5

(a): 6.25 μM, (b): 12.5 μM, (c): 65 μM unlabeled peptide + 6.25 μM CF-peptide, (d): 65 μM unlabeled peptide + 12.5 μM CF-peptide

Table S2. Mean viability (%) data recorded in flow cytometry experiments.

Sample name (Fig. S5)	Mean viability (%)
Control	59.4
P(AA) (1.6525 μM)	63.6
P(AA) (3.125 μM)	65.1
P(AA) (6.25 μM)	67.2
P(AA) (12.5 μM)	63.7
P(AA) (25 μM)	69.5
P(SC) (1.6525 μM)	59.5
P(SC) (3.125 μM)	66.9
P(SC) (6.25 μM)	62.3
P(SC) (12.5 μM)	69.2
P(SC) (25 μM)	64.4
P(YY) (1.6525 μM)	64.5
P(YY) (3.125 μM)	62.5
P(YY) (6.25 μM)	61.9
P(YY) (12.5 μM)	63
P(YY) (25 μM)	62.8
cP(AA)_P(YY) (1.6525 μM)	66.9
cP(AA)_P(YY) (3.125 μM)	60.8
cP(AA)_P(YY) (6.25 μM)	66.5
cP(AA)_P(YY) (12.5 μM)	64.2
cP(AA)_P(YY) (25 μM)	66.1
cP(SC)_P(YY) (1.6525 μM)	66.1
cP(SC)_P(YY) (3.125 μM)	63.5
cP(SC)_P(YY) (6.25 μM)	63.4
cP(SC)_P(YY) (12.5 μM)	60.4
cP(SC)_P(YY) (25 μM)	60.1
P(short)_P(YY) (1.6525 μM)	66.9
P(short)_P(YY) (3.125 μM)	65.8
P(short)_P(YY) (6.25 μM)	59.4
P(short)_P(YY) (12.5 μM)	63.2
P(short)_P(YY) (25 μM)	60.5
scr_P(AA_YY) (1.6525 μM)	57.9
scr_P(AA_YY) (3.125 μM)	57.9
scr_P(AA_YY) (6.25 μM)	62.4
scr_P(AA_YY) (12.5 μM)	61.1
scr_P(AA_YY) (25 μM)	66
**Sample name (Fig. S7)**	**Mean viability (%)**
Control	86.4
P(AA)	86.1
P(SC)	88.6
P(YY)	88.7
cP(AA)_P(YY)	87.8
cP(SC)_P(YY)	89.2
P(short)_P(YY)	83.0
scr_P(AA_YY)	84.0

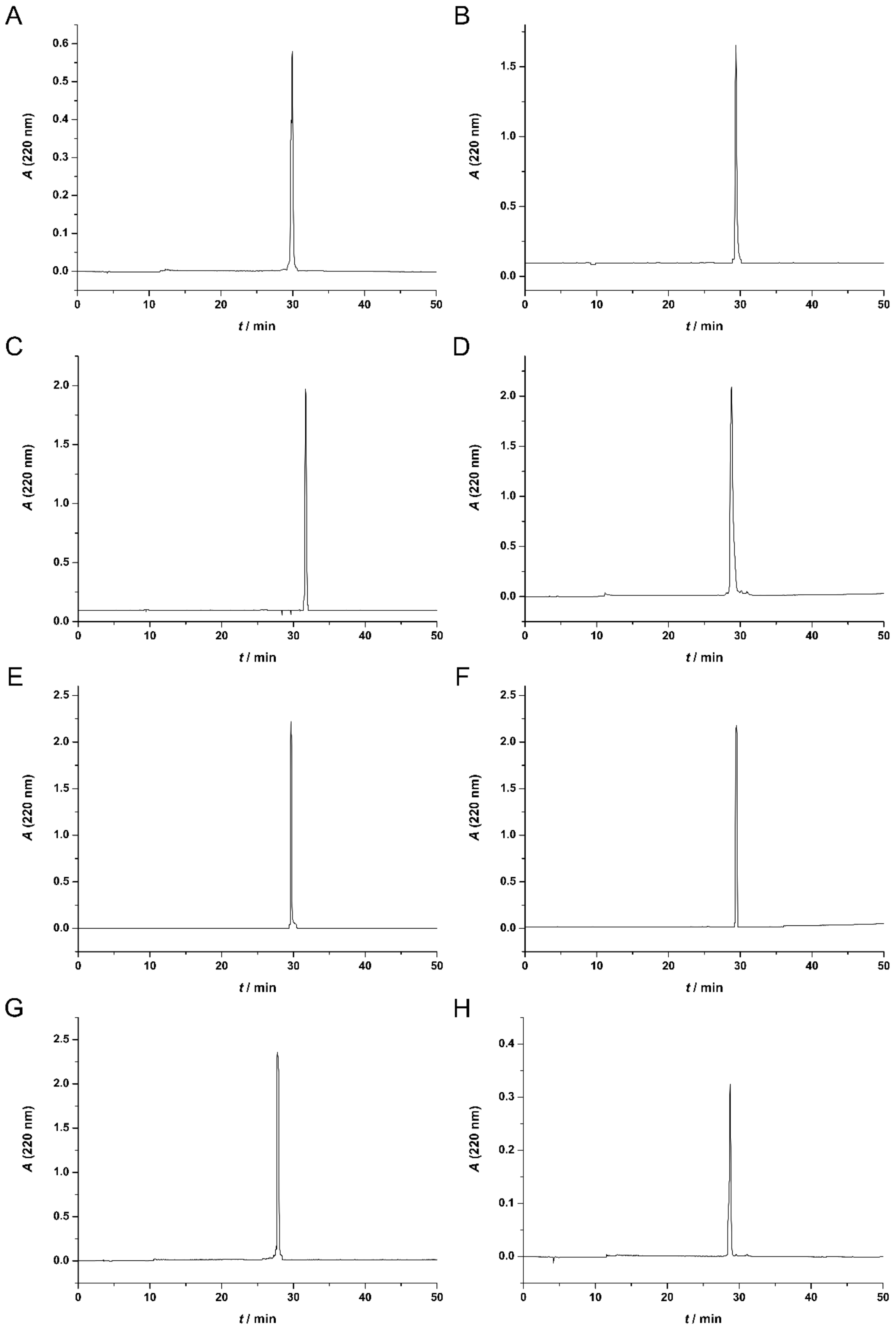

Figure S1. RP-HPLC profiles of conjugates (**A**) P(CC), (**B**) P(CGC), (**C**) P(CGGC), (**D**) P($C_{(Acm)}C_{(Acm)}$), (**E**) P(CS), (**F**) P(SC), (**G**) P(SS) and (**H**) P(AA).

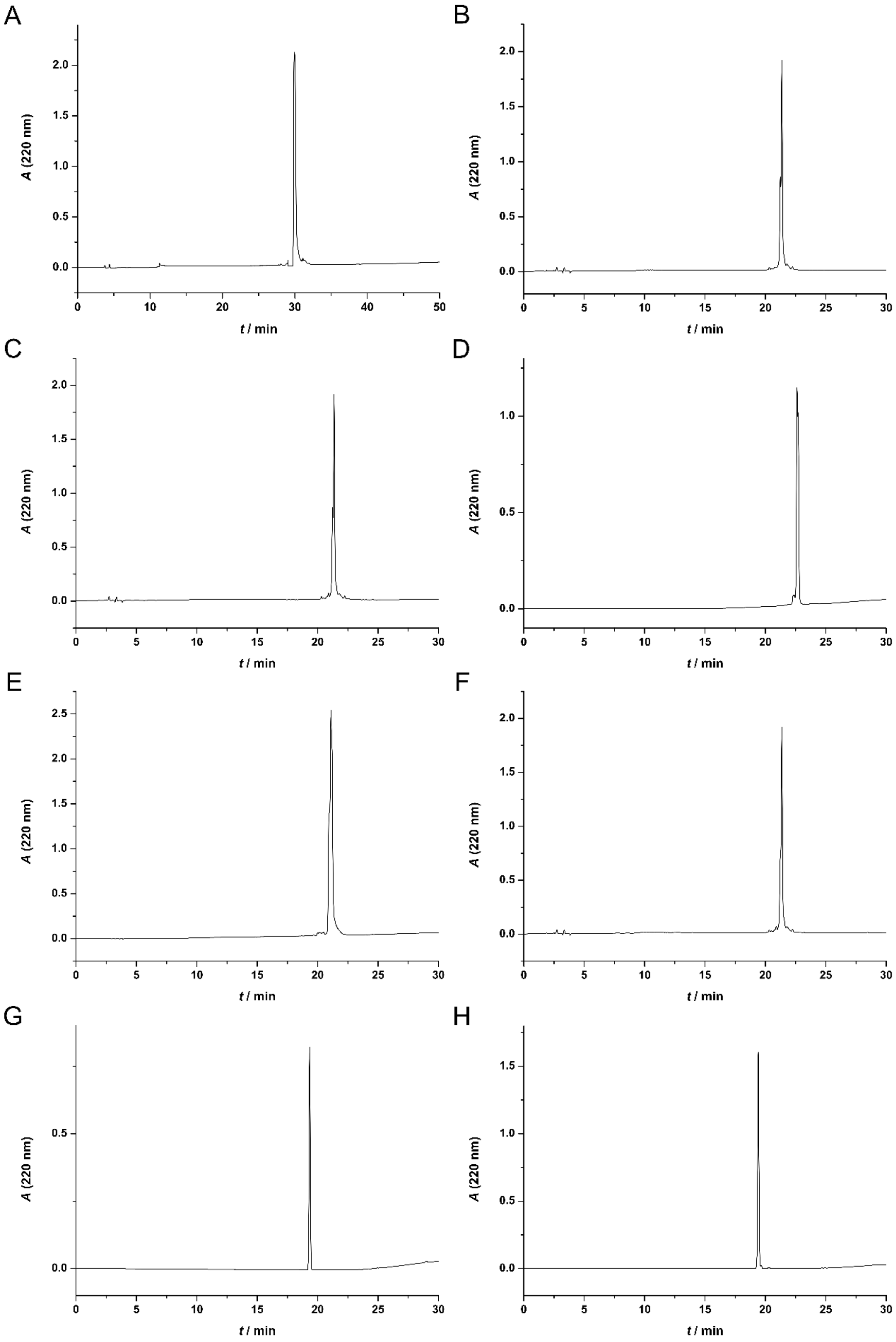

Figure S2. RP-HPLC profiles of conjugates (**A**) P(YY), (**B**) cP(AA)_P(YY), (**C**) cP(SC)_P(YY), (**D**) P(short)_P(YY), (**E**) scrP(AA_YY), (**F**) cP(YY)_P(AA), (**G**) Unlabeled cP(AA)_P(YY) and (**H**) Unlabeled cP(SC)_P(YY).

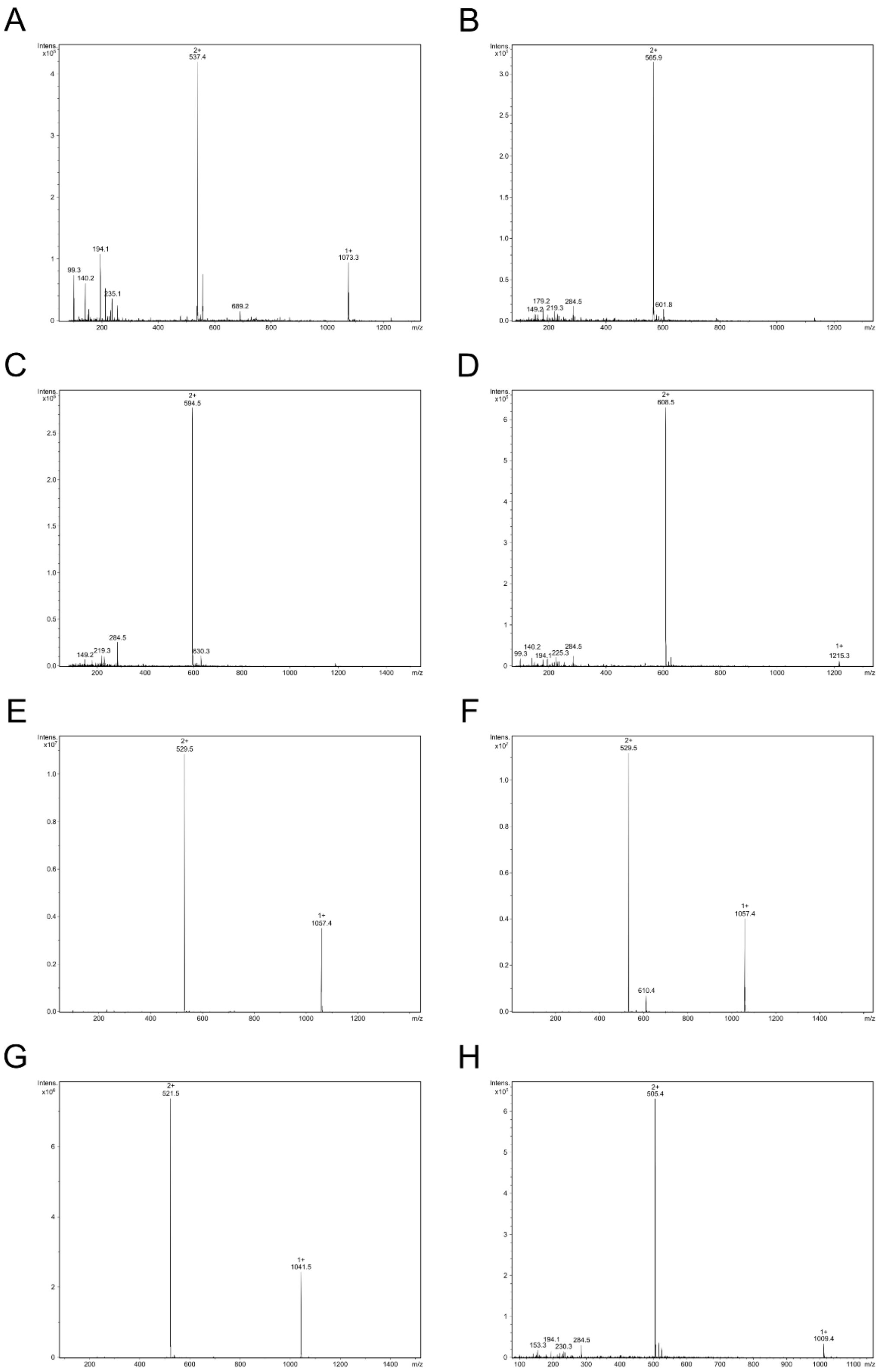

Figure S3. ESI-ion trap mass spectra of conjugates (**A**) P(CC), (**B**) P(CGC), (**C**) P(CGGC), (**D**) P($C_{(Acm)}C_{(Acm)}$), (**E**) P(CS), (**F**) P(SC), (**G**) P(SS) and (**H**) P(AA).

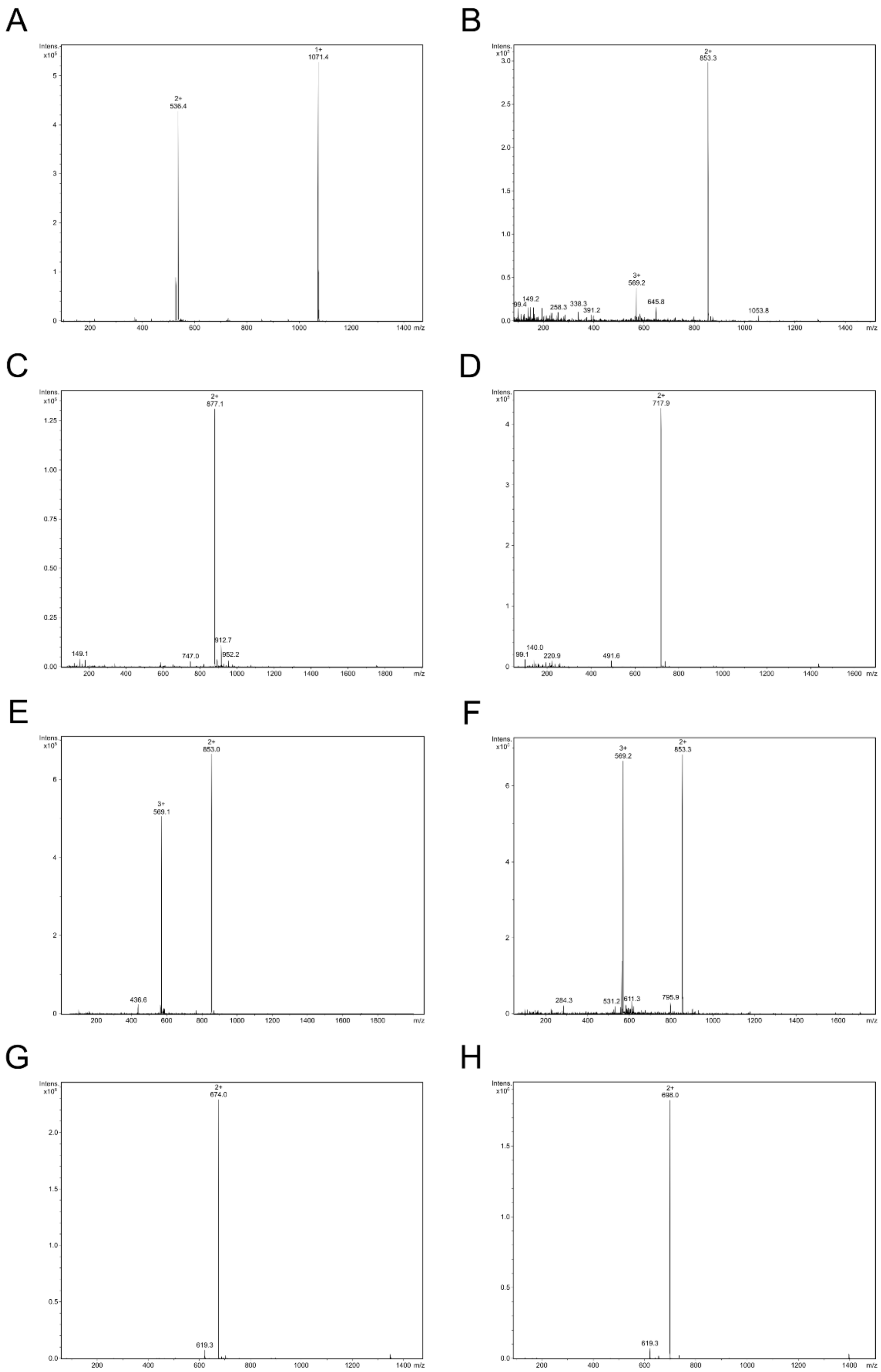

Figure S4. ESI-ion trap mass spectra of conjugates (**A**) P(YY), (**B**) cP(AA)_P(YY), (**C**) cP(SC)_P(YY), (**D**) P(short)_P(YY), (**E**) scrP(AA_YY), (**F**) cP(YY)_P(AA), (**G**) Unlabeled cP(AA)_P(YY) and (**H**) Unlabeled cP(SC)_P(YY).

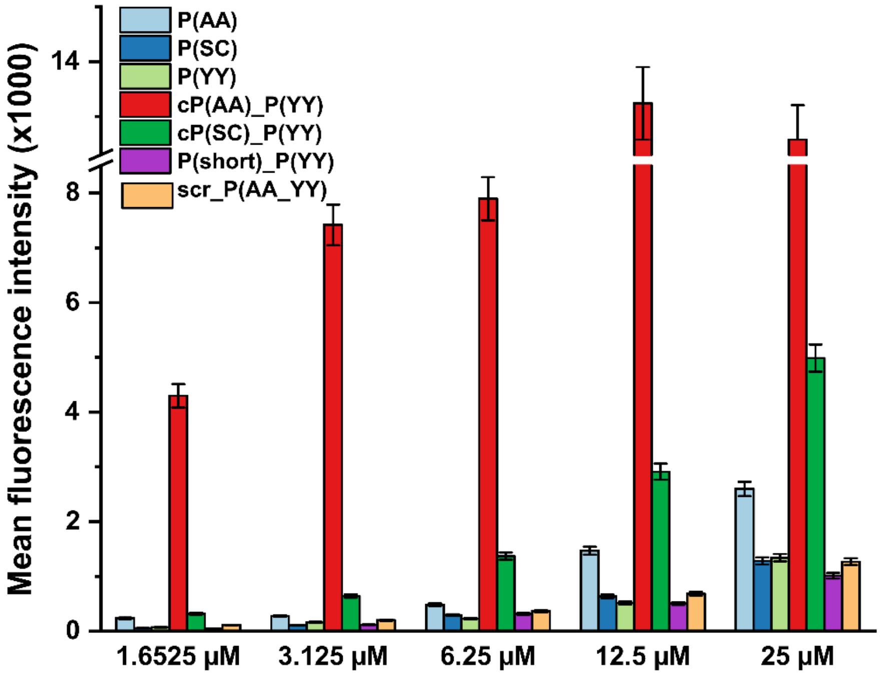

Figure S5. Concentration dependent cellular uptake profile of CF-labeled HER2 binding peptides using MDA-MB-453 breast cancer cells. Flow cytometry was measured after 3 h incubation by a BD LSR II flow cytometer, mean and standard deviation of mean fluorescence intensity using two parallels are depicted, the experiments were repeated twice.

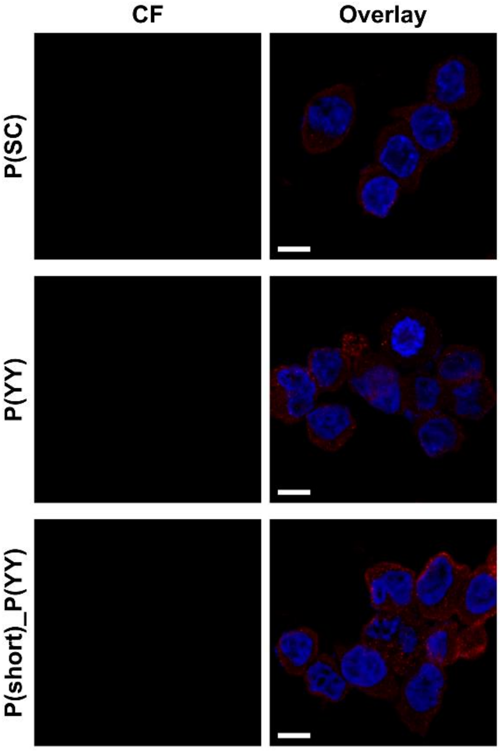

Figure S6. Cellular localization study of P(SC), P(YY) and P(short)_P(YY) in MDA-MB-453 cells using confocal microscopy. Cells were fixed after 90 min incubation with the peptides (25 μM, green). HER2 was detected with anti-HER2 antibody and TRITC-conjugated secondary antibody (red). Nuclei are stained with DAPI (blue). Zeiss LSM 710 system was used for image acquisition. Scale bars represent 10 μm.

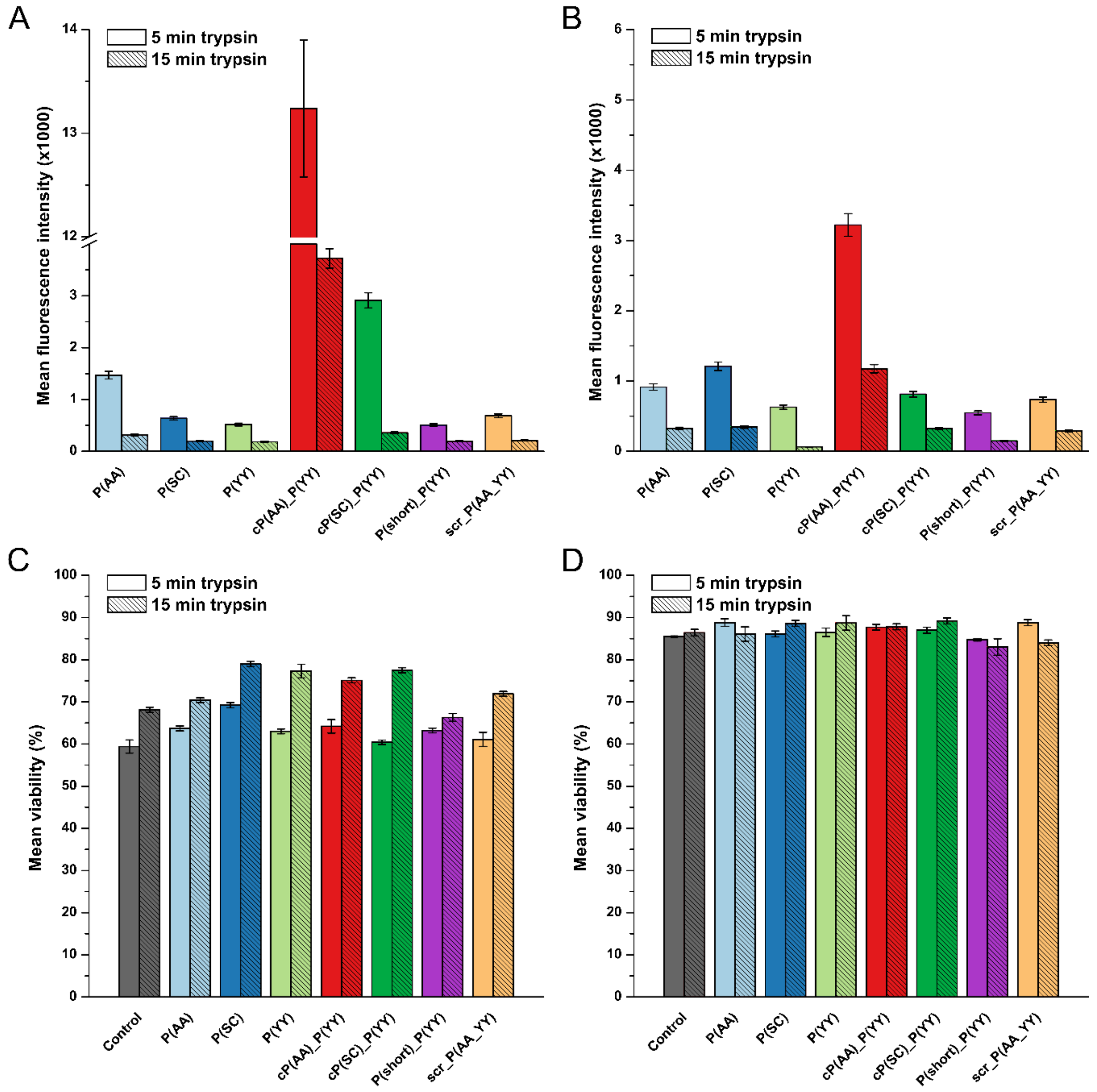

Figure S7. Effect of increased trypsinization time on cellular uptake of CF-labeled peptides by MDA-MB-453 and MDA-MB-435 Brain cells (**A** and **B**, respectively), along with the mean viability data recorded (**C** and **D** for MDA-MB-453 and MDA-MB-435 rain cells, respectively). Cells were incubated with the conjugates (12.5 μM) for 3 h. Flow cytometry was measured by a BD LSR II flow cytometer, mean and standard deviation of mean fluorescence intensity values using two parallels are depicted, the experiments were repeated twice.

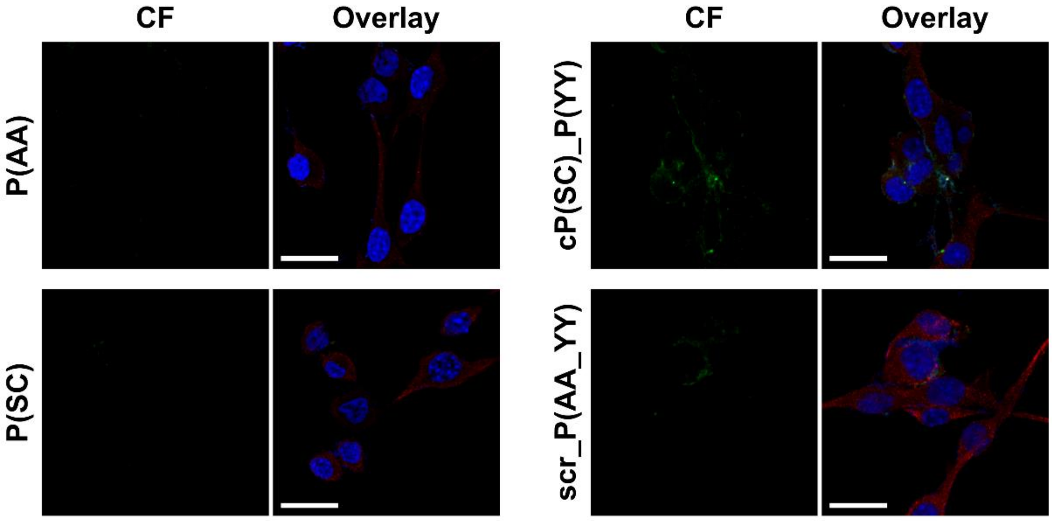

Figure S8. Cellular localization study of P(AA), P(SC), cP(SC)_P(YY) and scr_P(AA_YY) in MDA-MB-435 Brain cells using confocal microscopy. Cells were fixed after 90 min incubation with the peptides (25 μM, green). HER2 was detected with anti-HER2 antibody and TRITC-conjugated secondary antibody (red). Nuclei are stained with DAPI (blue). Zeiss LSM 710 system was used for image acquisition. Scale bars represent 20 μm.

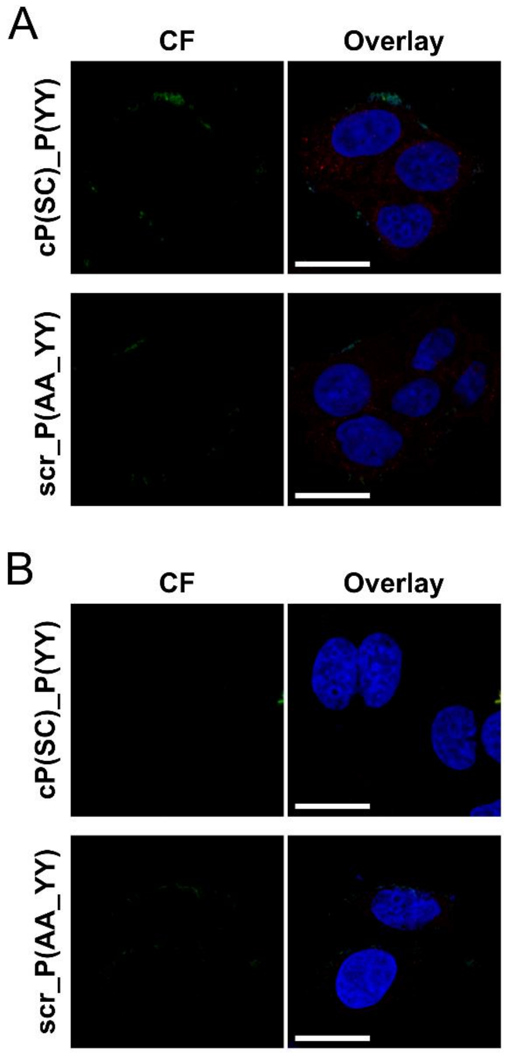

Figure S9. Cellular localization study of cP(SC)_P(YY) and scr_P(AA_YY) in MCF-7 (**A**) and HCC1143 cells (**B**). Cells were fixed after 90 min incubation with the peptides (25 µM, green). HER2 was detected with anti-HER2 antibody and TRITC-conjugated secondary antibody (red). Nuclei are stained with DAPI (blue). Zeiss LSM 710 system was used for image acquisition. Scale bars represent 20 µm.
